# Detection and Evaluation of Spatio-Temporal Spike Patterns in Massively Parallel Spike Train Data with SPADE

**DOI:** 10.3389/fncom.2017.00041

**Published:** 2017-05-24

**Authors:** Pietro Quaglio, Alper Yegenoglu, Emiliano Torre, Dominik M. Endres, Sonja Grün

**Affiliations:** ^1^Jülich Research Centre, Institute of Neuroscience and Medicine (INM-6) and Institute for Advanced Simulation (IAS-6), JARA Brain Institute IJülich, Germany; ^2^Chair of Risk, Safety and Uncertainty Quantification, ETH ZurichZurich, Switzerland; ^3^Risk Center, ETH ZurichZurich, Switzerland; ^4^Theoretical Neuroscience Group, Department of Psychology, Philipps-UniversitätMarburg, Germany; ^5^Theoretical Systems Neurobiology, RWTH Aachen UniversityAachen, Germany

**Keywords:** spike patterns, data mining, multiple testing, cell assemblies, higher-order correlations, spike synchrony

## Abstract

Repeated, precise sequences of spikes are largely considered a signature of activation of cell assemblies. These repeated sequences are commonly known under the name of spatio-temporal patterns (STPs). STPs are hypothesized to play a role in the communication of information in the computational process operated by the cerebral cortex. A variety of statistical methods for the detection of STPs have been developed and applied to electrophysiological recordings, but such methods scale poorly with the current size of available parallel spike train recordings (more than 100 neurons). In this work, we introduce a novel method capable of overcoming the computational and statistical limits of existing analysis techniques in detecting repeating STPs within massively parallel spike trains (MPST). We employ advanced data mining techniques to efficiently extract repeating sequences of spikes from the data. Then, we introduce and compare two alternative approaches to distinguish statistically significant patterns from chance sequences. The first approach uses a measure known as conceptual stability, of which we investigate a computationally cheap approximation for applications to such large data sets. The second approach is based on the evaluation of pattern statistical significance. In particular, we provide an extension to STPs of a method we recently introduced for the evaluation of statistical significance of synchronous spike patterns. The performance of the two approaches is evaluated in terms of computational load and statistical power on a variety of artificial data sets that replicate specific features of experimental data. Both methods provide an effective and robust procedure for detection of STPs in MPST data. The method based on significance evaluation shows the best overall performance, although at a higher computational cost. We name the novel procedure the spatio-temporal Spike PAttern Detection and Evaluation (SPADE) analysis.

## 1. Introduction

An open question in neuroscience is whether neuronal activity is organized in spatio-temporal patterns (STPs) of millisecond-precise spikes to represent and process information. Theoretical studies have shown that input spikes arriving synchronously at a neuron are most effective in generating output spikes, making the neuron behave like a coincidence detector (Abeles, 1982). The existence of coincidence detectors was shown in a number of experimental studies (e.g., Roy and Alloway, 2001; Bender et al., 2006; Fino et al., 2010). Different types of network models have been suggested that build on this fact to represent and process information. The synfire chain model, suggested by Abeles (1991), is composed of layers of groups of neurons that are feed-forward connected with large convergence and divergence, typical for cortical connectivity. If a group of neurons is activated simultaneously, synchronous activity is elicited and propagates to the next group of neurons, where it arrives simultaneously due to identical propagation delays. This group in turn sends synchronous spikes to the next and so on, such that volleys of synchronous spikes travel through the chain-like structure. This propagation is stable for a large range of parameters, such as the number of active neurons or the temporal precision (Diesmann et al., 1999). Bienenstock (1995) suggested a more general model, the synfire braid, which also builds on coincidence detectors but does not require the propagation delays between successive groups of neurons to be identical. Each neuron in the model still receives an abundance of synchronous spikes, but these may be generated at different times and yet arrive synchronously at the same target neuron due to different, compensating propagation delays. Izhikevich (2006) later rediscovered this idea under the name polychrony and proposed practical implementations of braid models. Common to both the synfire chain and the synfire braid/polychrony models is the fact that the activity they produce is characterized by specific STPs, which reoccur upon re-activation of the same network (chain/braid). The organization of the cortex may well support these or similar types of processing schemes that exploit spike coordination at a fine (millisecond precise) temporal scale, as opposed to or alongside with temporally loose rate-based coding schemes (Kumar et al., 2010; Ainsworth et al., 2012). In an electrophysiological study on few simultaneously recorded neurons, Prut et al. (1998) showed the occurrence of millisecond-precise STPs beyond the level expected on the basis of the neuronal firing rates, computed instead on a slower time scale of tens or hundreds of milliseconds. These patterns also showed tuning to behavior. Nevertheless, the existence of time-coding schemes in networks of several tens to hundreds of neurons remains debated due to the long-standing lack of data and of analysis tools suited for this investigation.

Recent progress in electrophysiological recording technology has enabled the simultaneous recording of hundred or more neurons, thus, increasing the chance to sample cells involved in complex dynamics, for instance those produced by synfire chains and braids. Analysis tools are, therefore, needed to identify this complex dynamics within such large data sets. Tools developed in former studies for the analysis of STPs in small populations of neurons (e.g., Prut et al., 1998; Nadasdy et al., 1999) scale poorly with the size of the data and thus cannot be applied to massively parallel spike trains (MPST). The core problem lies in the number of possible interactions (i.e., correlations in the spiking activity, here STPs) that need to be investigated without a-priori information on the structure of the network (as typical for experimental data). The number of patterns grows exponentially with (i) the total number of neurons observed, (ii) the pattern size (number of composing spikes: 2, 3, …) investigated, and (iii) the temporal relationships (lags between spikes of a pattern) one aims to focus on (e.g., synchrony or specific temporal sequences). The occurrences of each of these patterns have to be counted, and non-chance patterns have to be distinguished from chance patterns based on properties such as the number of composing spikes or the number of pattern repetitions. These factors lead to computational issues—even just storing all patterns and their occurrence counts in memory on a desktop computer may be impossible, and their analysis may be computationally not affordable. Furthermore, a severe multiple testing problem arises, i.e., the huge number of statistical tests may lead to a large amount of false positives or of false negatives after standard statistical corrections.

A number of techniques have been introduced in the last decade to analyze correlations in MPST by circumventing these issues. Population measures for synchrony detection (Grün et al., 2008; Louis et al., 2010; Staude et al., 2010a,b) have been introduced to assess the presence and magnitude (i.e., the order) of synchrony in MPST, without resolving individual spike patterns or groups of neurons possibly responsible for these correlations. Conversely, maximum entropy methods (Schneidman et al., 2006; Shlens et al., 2006; Pillow et al., 2008; Tang et al., 2008) have been successfully applied to identify groups of neurons participating in synchronous firing, at the expense of the correlation order, which can be analyzed (typically involving few neurons in the population). Shimazaki et al. (2012) introduced an analysis method that identifies higher-order correlation and their order based on information geometry and a state space approach, but is limited to a small number of neurons. For large number of simultaneously recorded neurons, previous studies on large data sets have typically limited the search to an heuristically determined subset of candidate patterns based on pairwise correlations (Gansel and Singer, 2012) or to patterns with a limited selection of specified inter-spike intervals (see Ayzenshtat et al., 2010, for a methodology applied to data from voltage sensitive dye imaging) or derived statistical tests for patterns significance on restricting assumptions, such as Poissonianity of firing rates (Abeles and Gerstein, 1988; Russo and Durstewitz, 2017).

To overcome these limitations, Torre et al. (2013) developed a method, named SPADE (synchronous pattern detection and evaluation), to assess the presence of statistically significant patterns of synchronous spikes. This approach should not to be confused with a sequential pattern mining algorithm, which shares the same acronym (Zaki, 2001). SPADE exploits frequent item set mining techniques (see e.g., Borgelt, 2012) to remove those spike patterns (usually the majority), which either occur too infrequently to be of interest in a subsequent statistical analysis (according to the criteria defined by the investigator) or which are trivially explained by other patterns in the data and, therefore, convey no additional information. SPADE retains only frequent, non-trivial patterns, and further assesses their statistical significance by a hierarchy of tests. This approach greatly diminishes the number of total tests to a level which is easily handled with standard statistical corrections, such as False Discovery Rate (Benjamini and Hochberg, 1995). Using SPADE on motor cortex spike train data from behaving monkeys, we demonstrated in Torre et al. (2016b) the presence of groups of excess synchronous spike patterns in populations of up to 150 simultaneously recorded neurons and demonstrated the specificity of these patterns to different motor tasks and different behavioral epochs therein. Differently from previous techniques, the analysis covers correlations of any order and resolves their neuronal composition.

In this study, we provide an extension of SPADE to spatio-temporal patterns, i.e., patterns not restricted to synchronous spiking. The resulting methodology has, thus, the potential of identifying STPs like those produced by the synfire chain or synfire braid model or by other expressions of assembly processing that rely on precise spike time coordination. Due to the temporal dimension taken into account here, the analysis of STPs has to deal with a much (possibly orders of magnitude) larger number of possible patterns compared to the analysis of spike synchrony. In Yegenoglu et al. (2016), we addressed this problem by a new approach based on Formal Concept Analysis (FCA; see Ganter and Wille, 1999) to efficiently find STPs in MPST data, count their occurrences, and evaluate their stability (see Kuznetsov, 2007) as a measure for non-randomness. We successfully applied the method to artificial test data of up to 50 parallel spike trains with an average firing rate of about 15 Hz each simulated for 1 s. The analysis was computationally too expensive to be applied on larger data sets.

Here, we improve the methodology introduced in Yegenoglu et al. (2016) in three respects. First, we use frequent item set mining on a suitably restructured format of the data as an equivalent but computationally more efficient alternative to currently available FCA algorithms. This shift of paradigm makes the method equivalent to SPADE from a procedural point of view. Second, we approximate exact stability with the Monte-Carlo approach suggested by Babin and Kuznetsov (2012), which reduces the cost of stability computation (previously the runtime bottleneck) by several orders of magnitude. This also allows us to compute different types of pattern stability and to develop different criteria to filter patterns on the basis of these types improving further the performance of pattern detection. Third, we extend to STPs the evaluation of pattern significance originally introduced in Torre et al. (2013) and compare it with the approach based on pattern stability.

Section 2 presents the various steps of our novel method and links them to previous methods, mainly to the work we presented in Yegenoglu et al. (2016) and Torre et al. (2013). Section 3 compares the performance of the stability-based and significance-based (SPADE) approaches for patterns filtering, and provides selection criteria for candidate patterns. We demonstrate the efficacy of the extended SPADE method in detecting STPs, while largely avoiding false positive detections in simulated MPST with different features typical for electrophysiological data, such as firing rates varying over time and across neurons. Finally, Section 4 discusses the advantages of SPADE over existing techniques for the analysis of correlations in MPST and proposes future studies.

## 2. Methods

The problem we are concerned with in this work is the extraction of spatio-temporal spike patterns in massively parallel spike trains and the classification of these STPs into those that occur reliably and those which do not, i.e., non-chance vs. chance events. Here we first review a state-of-the-art method based on Formal Concept Analysis (FCA) which we recently introduced to address this problem, and then we improve this method in various respects. The following sections describe the three main steps of the novel method, namely pattern extraction (2.1), identification of reliable patterns by means of various stability measures (2.2), and statistical assessment of pattern significance (2.3).

### 2.1. Extracting non-trivial patterns from large-size data

**Figure 1:**
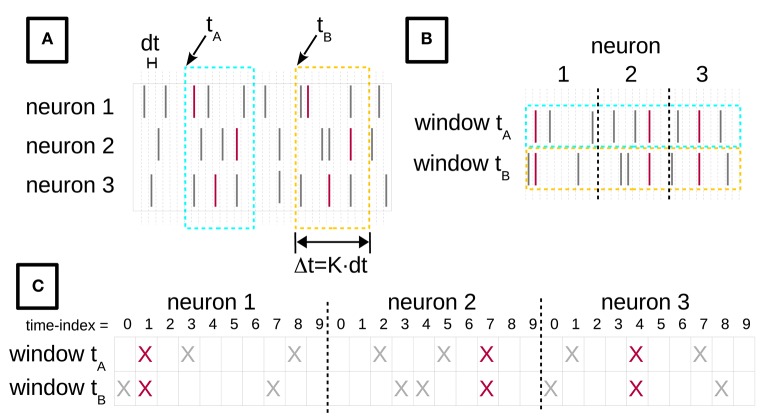
**Turning spike recordings from multiple neurons into a formal context**. **(A)** We discretize the time axis of parallel neuronal recordings from multiple neurons into bins of duration *dt*. The lines indicate spikes at their time of occurrence. The red spikes are members of an STP, whereas gray spikes belong to the background. We then chop time windows of length Δ*t* = *K* × *dt* out of the recorded data stream. **(B)** The window contents are then concatenated, for each neuron horizontally. **(C)** Each spike of **(B)** is replaced by a cross, yielding the incidence table representation of a formal context. The objects of this formal context are the time windows, indexed by their start time. The attributes are the spike time-indexes relative to the window start, combined with the neuron identities. The figure is adapted from Yegenoglu et al. (2016).

In our setting, an STP is defined as a pattern of spikes, emitted from a given collection of neurons, that have the same temporal relationship with each other across different occurrences. As an example, the red lines in Figure 1A form an STP composed of three spikes from neurons (in temporal order) 1, 3, and 2. Given *N* neurons, there can be up to 2^*N*^ sets of neurons participating in such a pattern. Each of them can form patterns with infinitely many different inter-spike intervals. Discretizing time into bins of short length (e.g., *dt* = 1 ms), as done in Figure 1A, makes the number of combinations finite for a finite-sized data set, but still immense even for relatively short recordings and few parallel neurons. Thus, the total number of possible STPs is too large for an exhaustive search. Of these, however, only a minority occurs multiple times (is *frequent*) and cannot be extended without sacrificing occurrences (is *closed*). Only frequent, closed patterns are of interest to us. Next, we introduce Formal Concept Analysis (FCA) for extracting closed frequent patterns from large data sets, adapting the methodology to parallel spike train data, and then relate Frequent Item set Mining (FIM) to FCA.

#### 2.1.1. Formal concept analysis (FCA)

A mathematical formalization of closed patterns was provided earlier by Wille (1982) in the theory of Formal Concept Analysis, whose basic notions we now introduce. Following the standard definitions (Ganter and Wille, 1999), we define a formal context as a triple ℂ = (*G, M, I*) comprising a set of formal objects *G*, a set of formal attributes *M*, and a binary relation *I* between the objects in *G* and the attributes in *M*. In our application, *G* contains the start times *t* of space-time windows (see Figure 1) and *M* the spike coordinates *m* in such windows. If (*t, m*) ∈ *I* with *t* ∈ *G*, *m* ∈ *M*, then window *t* contains a spike at *m*. We denote the set of all attributes shared by a set of objects *A* ⊆ *G* as *A*′ = {*m* ∈ *M*|∀*t* ∈ *A* : (*t, m*) ∈ *I*}, i.e., *A*′ is the set of all attributes *m* such that for all objects *t* in *A*, there is an entry in *I* which contains both *t* and *m*. Likewise, the set of all objects which have all the attributes in *B* ⊆ *M* is *B*′ = {*t* ∈ *G*|∀*m* ∈ *B* : (*t, m*) ∈ *I*}. A formal concept is a tuple (*A, B*) with extent *A* ⊆ *G* and intent *B* ⊆ *M* such that *A*′ = *B* and *B*′ = *A*. Let 𝔅(ℂ) be the set of all concepts of ℂ.

To gain an intuitive understanding of these definitions, consider the example in Figure 1. Figure 1A depicts spike recordings from three neurons and two space-time windows labeled by their starting times *t*_*A*_ and *t*_*B*_, i.e., *t*_*A*_, *t*_*B*_ ∈ *G*. The vertical ticks denote spikes. The red ticks are spikes that form an STP, whereas the gray ticks represent background spikes. The attributes in *M* are pairs (neuron index, time index) of the neuron from which a given spike was recorded, and of the spike time index relative to the window's start. Here, the red ticks correspond to the attribute set *H* = {(1, 1), (2, 7), (3, 4)} in both windows.

A standard way of depicting the relation *I* in the FCA literature is an incidence table (c.f. Ganter and Wille, 1999). Figure 1 illustrates the process of constructing such an incidence table from spike data. In Figure 1A we discretize the time axis into contiguous bins of duration *dt*. Then, we slide a time window of duration Δ*t* = *K* × *dt* across these data, in increments of *dt*. *t*_*A*_ and *t*_*B*_ in Figure 1A indicate two instances of this sliding window. The bin width *dt* is chosen depending on the resolution of the recording device and on the analysis needs, and determines the precision at which spatio-temporal patterns are resolved. We set *dt* = 1 ms throughout this paper, which ensures that there is at most one spike from the same neuron in each bin. The number of time bins *K* per window is selected on the basis of the expected maximal duration of an STP. We choose *K* as explained below. The incidence table representation of *I* is constructed by horizontally concatenating the contents of each window instance (Figure 1B), and finally converting the spikes into crosses (Figure 1C). The resulting table will have *N* × *K* columns.

We can then apply FCA to compute 𝔅(ℂ). The intents of the concepts in 𝔅(ℂ) are candidate STPs, while their extents are the sets of time indexes of window starts. For example, the data depicted in Figure 1A contain a concept *U* = (*A, H*) with extent *A* = {*t*_*A*_, *t*_*B*_} and intent *H* = {(1, 1), (2, 7), (3, 4)}. Another concept in this example is *V* = (*E, F*), with *E* = {*t*_*A*_} and *F* = {(1, 1), (1, 3), (1, 8), (2, 2), (2, 5), (2, 7), (3, 1), (3, 4), (3, 7)}. By “candidates” we mean that the intents of the concepts in 𝔅(ℂ) comprise not only the STPs we are interested in, but also time-shifted version thereof. Consider the windows starting one bin before *t*_*A*_ and *t*_*B*_. They, too, contain the STP comprised of the three red spikes, but shifted by one time step, leading to the corresponding attribute set *Q* = {(1, 2), (2, 8), (3, 5)}, which will become the intent of a concept after FCA. Thus, for a chosen *K*, the intents of the concepts in 𝔅(ℂ) contain all shifted copies of an STP. These copies form an equivalence class, which we represent by the STP whose first spike falls into the first bin of the window.

#### 2.1.2. FP-growth as an equivalent alternative to fast-FCA

**Figure 2:**
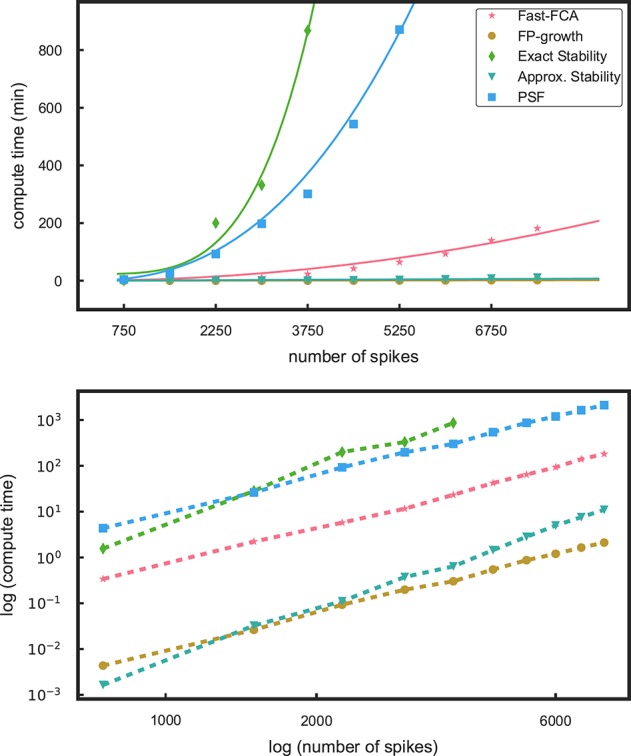
**Profiling results for different components of the methods**. In the top panel the runtimes as a function of the number of spikes in a data set are shown for pattern mining using fast-FCA (red, asterix) and FP-growth (brown, filled circles). We also compare the run times for the stability analyses, exact stability (green, diamonds) and approximate (aquamarine, triangles) and of PSF (blue, squares). The solid lines are fitted functions quantifying their characteristics. The bottom panel shows the same data in log-log scaling: the computational times follow approximately different power laws.

In Yegenoglu et al. (2016), we employed our pure Python implementation of the fast-FCA algorithm (Lindig, 2000). This algorithm creates the concepts in an order which simplifies the subsequent exact evaluation of stability used for isolating stable patterns from noise (see below). Unfortunately, the runtime of the computation of exact stability (see Roth et al., 2008) scales roughly with the fourth power of the number of spikes, as illustrated in Figure 2. This leads to a slow computation preventing the application to data sets of several tens of neurons. In Yegenoglu et al. (2016) we extrapolated the computation time to >60 days on a data set of 15 s duration composed of simultaneous recordings of 100 neurons. However, when one does not compute stability, or computes it approximately rather than exactly (see below), the concept order is not needed, as explained in Babin and Kuznetsov (2012). Modern FCA algorithms exist that only compute the concepts and are, therefore, considerably faster, such as In-Close (Andrews, 2009) which is currently the fastest one to our knowledge. Unfortunately, at the time of writing, the state-of-the-art *C* implementation of In-Close exited because of a memory overflow when we input our data. The *C* implementation solving this problem will be provided soon (Kodoga, personal communication).

Instead, we explored another option for implementing a faster search for concepts. We exploited a known correspondence between FCA and (closed) frequent item set analysis (Zaki and Ogihara, 1998; Pisková and Horváth, 2013): formal objects can be mapped onto transactions, formal attributes onto items, intents onto closed itemsets and extent sizes onto supports, see also Table 1. This allows us to compute the concepts based on the FP-growth algorithm known in the data mining community (Han et al., 2004). FP-growth is a frequent item set mining algorithm widely used to mine closed frequent item sets in large data sets. Specifically, we use a *C* implementation of FP-growth (Borgelt, 2012) to mine closed frequent patterns. We already used the algorithm in Picado-Muiño et al. (2013) to mine patterns of synchronous spikes in MPST data. The re-formatting of the data used here (“attribute scaling” in the terminology of FCA, see Ganter and Wille, 1999) allows us to extend the application of the FP-growth algorithm to the search for more general spike patterns, i.e., STPs.

**Table 1:** **Summary of terms often used in this paper**.

**Entity**	**Description**
Spatio-temporal pattern (STP)	Precise temporal sequence of spikes repeated more often than expected under the hypothesis of independent firing
Item set/attribute set	Set of spikes that form an STP
Pattern size/attribute set size	Number of spikes forming an STP
Occurrence times/object set	Set of time points at which an STP repeatedly occurs
Support/object set size	Number of occurrences of an STP
Closed item set/intent of formal concept	STP which is maximal in time and space, i.e., no larger set of time windows contains the STP, and no spike could be added to the STP without having to give up at least one occurrence time window. Formally defined as the pair of STP intent and STP extent. Extracted from parallel spike train data by the FP-growth/fast-FCA algorithm.
Support of closed item set/extent size	Number of occurrences of a STP which is maximal in time and space
Closed frequent item set/Intent of frequent formal concept	STP which is maximal in time and space, and occurs at least a given number of times.
Signature (*z, c*)	Pair of parameters (*z* = pattern size, *c* = support), that characterize each concept and that are tested for significance with PSF
Stability	Measure that quantifies how reliably a pattern repeats identically across all its repetitions
*P*-value spectrum	Matrix whose entries *z, c* contain the *p*-values of pattern signatures (*z, c*), evaluated by PSF

*When two alternative terms appear in the left column, the first one is from frequent item set mining terminology and the second one from formal concept analysis terminology*.

#### 2.1.3. Closed vs. non-chance patterns

By use of fast-FCA/FP-growth algorithms, formal concepts/closed frequent item sets can be efficiently collected. Closed patterns can be understood as patterns, which are not trivial subsets of other patterns in the data, and which, therefore, may convey information not stored in any superset of them. However, not all closed patterns are necessarily of interest. Indeed, virtually any data set of simultaneous point processes contains closed patterns, even when the processes are completely independent of each other. Thus, many (usually the large majority) of closed patterns are merely chance events. A critical task that remains to be solved is, therefore, that of identifying non-chance patterns among the multitude of closed ones.

Several approaches can be taken to draw this assessment, depending on the features of the patterns used to discriminate interesting from non-interesting STPs. Simple pattern filtering criteria are often based on pattern size (intent size) and pattern occurrence count (extent size), see e.g., Prut et al. (1998). These criteria are motivated by the following observations. First, the larger a pattern is the less likely it will occur by chance if spikes are independent events (Grün et al., 2002b). Likewise, under this independence assumption, the probability that a chance pattern has a given occurrence count decreases with increasing size (Grün et al., 2008). We found that rejecting all patterns with less than three spikes or less than three occurrences massively reduces the false positive pattern detection in our data. Another classical approach in the FCA community is to evaluate the (intensional) stability of a pattern (Kuznetsov, 2007), which loosely speaking can be understood as the tendency of a pattern to be an intent among any subset of windows where the pattern occurred. Stable patterns are of interest because they are unlikely to be produced by independent processes or neurons. Another approach consists in evaluating the statistical significance of the patterns found and in retaining only those patterns, which are not to be expected in the data given other statistics, such as the firing rates of the individual neurons (Grün et al., 2002a). This approach is common when testing the two alternative hypotheses of temporal coding (based on millisecond precise coordination of spikes among cell assemblies) vs. rate coding (based on temporally less precise spike coordination and characterized by the rate profiles of the individual neurons). The next two sections are dedicated to reviewing the approach based on stability computation and the other based on evaluation of statistical significance and to integrate them into the analysis framework derived so far.

### 2.2. Filtering patterns by stability

Conceptual stability (Kuznetsov, 2007), both intensional and extensional, is a potential tool for separating chance patterns from non-chance STPs. In this section, we review the definition of conceptual stability, and we illustrate advantages and computational issues thereof. We also show how recently developed efficient Monte-Carlo techniques can be used to approximate stability and, thus, make it applicable to large-size data.

#### 2.2.1. Intensional stability

Given a formal context ℂ and the set 𝔅(ℂ) of all of its concepts, the intensional stability of a concept (*A, B*) ∈ 𝔅(ℂ) is defined by Roth et al. (2008) as

(1) $$\sigma (A,B)=\frac{\left|\{C\subseteq A|C^{\prime }=B\}\right|}{2^{\left|A\right|}}$$

where *C*′ is the set of all attributes shared by the objects in *C* (see also section 2.1.1). In words, the stability of an intent is the fraction of subsets of the extent (set of the indices of the starting points of time windows), which share the attributes of the intent (pattern). This can be viewed as a kind of cross-validation (Kuznetsov, 2007): a pattern has a high stability index if it is found as a concept intent in many time windows. In our earlier work (Yegenoglu et al., 2016), we computed the stability with the exact algorithm of Roth et al. (2008) and kept only those concepts whose stability exceeded a threshold. However, we found that this exact algorithm is too slow for application to large data sets due to its quadratic runtime scaling in the number of concepts.

#### 2.2.2. Approximation of stability

Hence, we employ a fast Monte-Carlo approximation of the stability suggested by Babin and Kuznetsov (2012). Instead of iterating through all subsets of an extent in the numerator of the right hand side of Equation (1) and checking whether the attributes shared by a given subset equal the intent, one performs this check only on a fixed number *Z* of randomly drawn extent subsets. The denominator is then replaced by *Z*.

#### 2.2.3. Extensional stability

By definition, intensional stability only accounts for the occurrence count of a pattern and not for its size. Therefore, its value is unaffected (see also Yegenoglu et al., 2016) by the number of spikes forming the pattern. This behavior is evident in the statistical evaluation results shown below. This feature is independent of the approach (exact or approximated) used to compute the stability. However, pattern size should play a role in determining whether a pattern is to be retained as a true pattern or rejected as a chance event. Indeed, more independent events (spikes) are less likely to re-occur in a specific temporal sequence by chance than fewer events. To account for this fact, we introduce here a filtering rule based on extensional stability, which accounts for the pattern composition (size) rather than the pattern occurrence count. Formally, extensional stability of a concept (*A, B*) is defined by exchanging extent and intent on the right hand side of Equation (1):

$$\sigma (A,B)=\frac{\left|\{C\subseteq B|C^{\prime }=A\}\right|}{2^{\left|B\right|}}.$$

Extensional stability can be calculated—as intensional stability—either in exact form or by approximation. A new filtering criterion can be devised for closed patterns based on extensional stability by retaining only those patterns whose extensional stability exceeds some pre-defined threshold.

#### 2.2.4. Choice of stability threshold

An issue that remains to be addressed is how to set the stability threshold(s) used to distinguish STPs from chance patterns. In Yegenoglu et al. (2016), we set the threshold for intensional stability to 0.6 following an heuristic approach, as this choice turned out to provide a good balance between FPs and FNs on a broad range of simulated data with various parameters. Real data, however, may need different thresholds depending on their size (number of neurons and/or duration), the firing statistics and other features of the spike trains. Because we are interested in using stability as a measure to determine which patterns are more stable than one would expect a chance pattern to be, an appropriate threshold should be such that the stability of patterns found in independent data, i.e., of chance events, would not cross the threshold. We, therefore, propose here to estimate the appropriate stability threshold from independent surrogates of the original data via the following Monte-Carlo approach.

First, surrogates of the original data that contain only chance patterns need to be generated in such a way that other features of the data characterizing the null hypothesis of independence (importantly, the firing rate profiles) are preserved. A variety of techniques exists to this end (see Grün, 2009; Louis et al., 2010). Among them, we opt for spike dithering, which moves each spike by a random amount (up to a few ms) around its original position. STPs occurring above chance level, if existing are, thus, destroyed, while firing rates - which are defined on a larger time scale—are almost unaffected. Second, we extract patterns from the surrogate data by use of FP-growth, compute their stability, and thereby derive the distribution of pattern stability under the null hypothesis. The stability threshold is finally set to a chosen upper quantile of the null distribution. In our settings, a single surrogate data set contains always several thousands of chance patterns and is, therefore, sufficient to obtain close estimates of small quantiles of the null distribution. We separately derive the thresholds θ_int_ and θ_ext_ for intensional and extensional stability, respectively.

### 2.3. Filtering patterns by statistical significance

An alternative to stability-based filtering to identify non-chance patterns among the closed frequent patterns extracted by FP-growth is to test the statistical significance of STPs directly. The null hypothesis of the test here is that the spike trains are mutually independent and no patterns exist in the data except for chance ones. The alternative hypothesis states that some patterns indeed occur too many times to be considered as chance events.

Testing the statistical significance of all closed frequent patterns one by one is not an option in applications to large-size data such as MPST data from tens or hundreds of neurons recorded simultaneously. Indeed, the immense amount of occurring patterns and, therefore, of tests to be performed raises severe multiple testing issues. We addressed this problem in the context of testing for synchronous spike patterns in Torre et al. (2013), where we developed an alternative statistical approach, here named SPADE (Synchronous PAttern Detection and Evaluation), that allows us to avoid such massive multiple testing. In that publication, we employed FP-growth to extract synchronous patterns, as we have done here for the more general case of spatio-temporal spike patterns. Thus, we aim to employ the statistical framework of SPADE to test for STPs. In the following we summarize the various steps of the SPADE analysis to assess pattern significance.

#### 2.3.1. Pattern spectrum filtering

The first component of SPADE for assessing the significance of closed frequent patterns found by FP-growth is Pattern Spectrum Filtering (PSF). Instead of testing individually each of the thousands of closed frequent patterns, statistical significance is assessed for patterns of same size *z* and same number of occurrences *c*, i.e., for each pattern signature (*z, c*). The probability of having a pattern with signature (*z, c*) under the null hypothesis H0 of independence is evaluated via a Monte-Carlo technique on surrogate data which are generated from the original data by dithering. By repeated generation of surrogates and counting closed frequent item sets we implement the null-hypothesis of independence. SPADE then determines the *p*-value of each signature (*z, c*) as the fraction of surrogates that contain closed frequent item sets with that signature, based on a large total number *K* of surrogates. Here, differently from what was necessary for the choice of the stability threshold, a large number of surrogates is needed, because each one contributes with only one instance (pattern of a certain signature (*z, c*) present or not) to the Monte Carlo sampling. Since multiple tests are performed (one per signature found in the original data), we correct the significance level α using the false discovery rate (FDR) correction (Benjamini and Hochberg, 1995). All patterns mined in the original data with signatures (*z, c*) that have a *p*-value smaller than the FDR corrected threshold are classified as statistically significant.

#### 2.3.2. Pattern set reduction

In Torre et al. (2013) we showed that the presence of repeated occurrences of a real pattern *A* tends to increase the significance of patterns resulting from the chance overlap of pattern *A* with background activity. In other words, PSF correctly rejects FPs entirely composed by chance patterns, but, in the presence of a real pattern, it tends to overestimate the significance of patterns resulting from chance overlap with background spikes. The reason is that the size *z* and/or the occurrence count *c* of these patterns are indeed not entirely due to chance, but are boosted by the presence of the real pattern beyond the chance level that PSF determined under H0.

Pattern Set Reduction (PSR), the last step of the SPADE analysis, aims at removing these FPs by testing the patterns filtered with PSF reciprocally for conditional significance. When testing for a pattern *A* given a sub-pattern *B* of *A* (such that *z*_*A*_ > *z*_*B*_ and *c*_*A*_ < *c*_*B*_), PSR re-assesses the significance of *A* through the *p*-value of the signature (*z*_*A*|*B*_ = *z*_*A*_ − *z*_*B*_ + *h, c*_*A*_) already stored in the *p*-value spectrum previously computed by PSF. *z*_*A*|*B*_ is a smaller value than *z*_*A*_, penalized by the presence of *B*. Similarly, *B* is re-tested conditioning on the presence of *A* by replacing its occurrence count *c*_*B*_ with *c*_*B*|*A*_ = *c*_*B*_ − *c*_*A*_ + *k*. *h* and *k* are correction factors accounting for the fact that the *p*-values of (*z*_*A*|*B*_, *c*_*B*_) and (*z*_*A*_, *c*_*B*|*A*_) are taken from the original *p*-value spectrum, which is calculated over all time bins rather than over the time bins only where *A* and *B* occur. In our study we set *h* = 0, *k* = 2, which proved to be a good heuristical choice in the validation of SPADE (see also Torre et al., 2013). If only (*z*_*A*|*B*_, *c*_*A*_) is significant, the method retains *A* and discards *B*, and vice versa if only (*z*_*B*_, *c*_*B*|*A*_) is significant. If both (*z*_*A*|*B*_, *c*_*A*_) and (*z*_*B*|*A*_, *c*_*B*_) are significant, both patterns are kept. If neither signature is significant, in light of the fact that PSF returned both and, therefore, at least one of the two patterns should be a true positive, PSR retains the pattern covering the largest number of spikes, i.e., the patterns with the largest *z* × *c* score. For patterns *A* and *B* that only partially overlap (*A* ∩ *B* ≠ ∅, *A* ⊄ *B* and *B* ⊄ *A*) the conditional tests are performed over the conditioning pattern *A* ∩ *B*.

## 3. Results

We presented above two different techniques to distinguish between chance patterns and selected STPs, based on stability measures and based on statistical significance of signatures (SPADE), respectively. Both of them take as input the concepts mined with FP-growth and return those that are statistically surprising because the assessed feature (stability or signature) is significantly larger for these patterns than for chance patterns. In this section we illustrate how each of the two methods performs, both in terms of computational time and of false positives and false negatives.

### 3.1. Computational efficiency

We first compare the computational efficiency of the components of the method by Yegenoglu et al. (2016) (fast-FCA, exact stability) to the proposed components introduced in the section above (FP-growth, approximate stability, PSF). Figure 2 shows the runtimes of these components applied to simulations of 50 parallel, mutually independent Poisson spike trains with a firing rate of 15 Hz each. The runtime of these various analyses components is evaluated on 10 data sets of different number of spikes, achieved by data sets of different duration, increasing from 1 to 10 s in steps of 1 s. The measured runtimes are marked by symbols, and their fitting curves are shown as solid lines in the same color. The profiling results were obtained on a compute cluster with 32 nodes, each consisting of a 2 × Intel Xeon E5 processor with 2.5 GHz processing speed and 8 × 16 GB DDR4 RAM.

In Yegenoglu et al. (2016), we made use of the fast-FCA algorithm introduced by Lindig (2000) after pre-processing the data as described in 2.1.1. The runtime behavior of the fast-FCA algorithm implemented in Python (same as used in Yegenoglu et al., 2016) and shown in Figure 2 (red) is fitted by a function which is quadratic in the number of spikes. Based on this function we extrapolate the runtime of larger data sets, in particular to the typical experimental data we aim to analyze, i.e., 100 parallel neurons with an average firing rate of 15 Hz of each neuron, recorded for 15 s and, thus, containing a total of 22,500 spikes on average. Mining the concepts in a data set of this size with this implementation of fast-FCA would take about 68 days of compute time. FP-growth (brown) is significantly faster and exhibits a significantly slower and linear trend. For a data set of the same size the runtime is 4.5 h instead. Thus, the speed up gained by using FP-growth instead of FCA enables the extraction of non-trivial patterns also from large-size data that were beyond the reach of our previous approach. Therefore, we decide to base our analysis on FP-growth.

The second step of our analysis is the computation of the stability (intensional and extensional stability) of all non-trivial patterns extracted by FP-growth, to filter out non trivial patterns (2.2). The stability can either be computed exactly or can be approximated by a Monte-Carlo approach (see 2.2.2). We show here only the result for the intensional stability, since the runtime for the calculation of extensional stability is approximately the same (not shown here). The runtime necessary to derive the stability as described in 2.2.4 is the sum of the time required to compute the stability on the original, empirical data set for each pattern and the time needed to compute its significance threshold. The latter requires the generation of a surrogate data set, the extraction of closed patterns, and the computation of their stability. Thus, the total runtime of our stability-based STP detection approach (see 2.2) is twice the time needed for the calculation of the stability, plus twice the time consumed by FCA (see 2.2.4). As shown in Figure 2, the computation of the exact stability (green) dominates the total runtime, increasing quartically with the number of spikes in the data. The approximate stability (aquamarine), in contrast, has a runtime which is several orders of magnitude smaller and shows a linear trend.

Overall, replacing FCA by FP-growth and replacing exact stability by approximate stability yields a compute time, which is about three orders of magnitude smaller and, thus, enables applications to data of unprecedented size.

The third and last step of the method that needs to be investigated in terms of runtime is the calculation of the statistical significance of the patterns by means of pattern spectrum filtering (PSF) and pattern set reduction (PSR). To derive the *p*-value for PSF for each signature, we need to generate at least 1,000 surrogate data, each of which requires to be analyzed by FP-growth to extract closed frequent patterns in order to build up the statistics for each signature. Therefore, PSF is quite compute time intensive (Figure 2, blue—for a data set of 22,500 spikes it would take about 1,366 days) if processed in a serial way as shown here. Trivial parallelization (i.e., parts of the analysis can be run independent from each other to save compute time) of the analysis program can be applied to PSF, which absorbs the majority of the computational load of the method and reduces severely the computational time. To this end, the FIM analysis on different surrogates can be distributed over different computing cores and run in parallel. The independent results (closed frequent patterns of each surrogate data set) are finally collected to compute the *p*-value spectrum. The PSR runtime is negligible (not shown here) since it is linear and it is applied only to significant patterns which is typically a small number as compared to the mined concepts. It does not directly depend on the total number of spikes but on the actual significant patterns. For this reason we do not consider it as a computational component that might determine the computational feasibility of SPADE.

### 3.2. Stochastic models for validation

The increased computational performance achieved by combining FP-growth and approximate stability calculation enables the application to larger data sets than previously possible. We are, therefore, interested in generating ground truth artificial data with comparable size and properties of data typically obtained in electrophysiological recordings. To this end, we follow the same approach taken in Yegenoglu et al. (2016) and generate data consisting of a superposition of independent background activity and repeated STPs. The background activity is modeled by a set of *N* = 100 parallel independent Poisson processes, each having a firing rate *r* which may be stationary or variable over time, and identical or different across neurons, and lasts for a total period *T* = 1 s. An STP occurrence is modeled as a temporal sequence of *z* spikes from the first *z* neurons (without loss of generality) and with a constant time lag of 5 ms between successive spikes. Multiple occurrences of the STP are realized by injecting the sequence at multiple, random times within the simulation interval [0, *T*].

**Figure 3:**
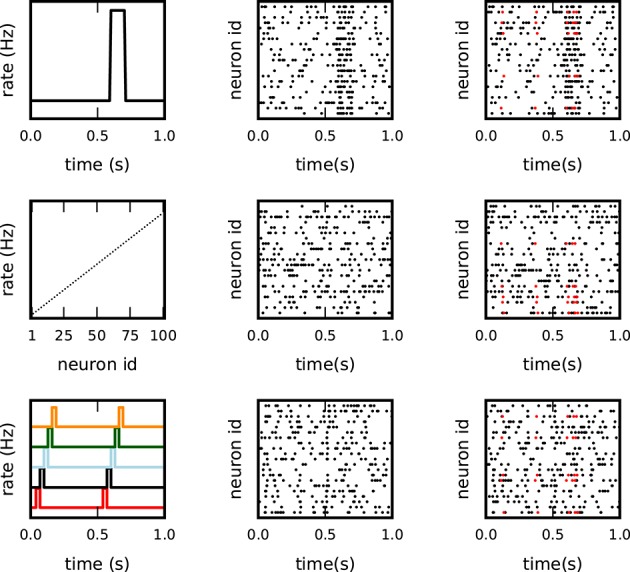
**Different background models for non-stationary and inhomogeneous data**. Each row illustrates one model of the test data: top: co-varying firing rates with a large rate step of all neurons; middle: inhomogeneous but stationary rate of each neuron; bottom: coherent short rate changes in subsets of neurons at consecutive time points. The columns show from left to right: the underlying rate profiles, an exemplifying raster plot of the spiking activity (one dot per spike), and a raster plot of the respective background activity enriched with *c* = 5 injections of an pattern of size *z* = 5 (spikes in red).

We first consider stationary data with three different constant firing rates *r* ∈ {15, 20, 25 Hz} for each of the 100 neurons. Then, we test the performance of the method for a variety of non-stationary data sets that mimic typical statistical features of experimental data and have the tendency to generate FPs in correlation analyses. In particular, we analyze artificial data that include three different types of rate non-stationarity or variability (see Figure 3):

1. Non-stationary firing rates over time by means of a sudden rate jump, coherent across all neurons (Figure 3, top panel): all neurons have the same firing rate, equal to 10 Hz in the intervals [0, 0.6 s] and [0.7, 1 s], and 60 Hz in the interval [0.6, 0.7 s];
2. Heterogeneity of the firing rates across neurons (Figure 3, middle panel): firing rates are stationary over time but different across neurons, and increase from 5 Hz (for the first neuron) to 25 Hz (for the last neuron) in steps of 0.2 Hz. The spike trains in which the patterns are later injected are randomly selected;
3. Short lasting, simultaneous, sequential rate jumps of subsets of neurons (Figure 3, bottom panel): the 100 neurons are grouped into 20 subsets of 5 neurons each. At two different time onsets (50 and 550 ms), the first group instantly changes its firing rate from a baseline level of 14 to 100 Hz for an interval of 5 ms. When group *i* goes back to baseline level group *i* + 1 experiences the same rate jump (5 ms later), *i* = 1, …, 19.

Models 1 and 2 were already used in Torre et al. (2013) to explore the sensitivity of SPADE to rate variability. The third model was introduced in Torre et al. (2016a) to validate another method, called ASSET, designed for the analysis of sequences of synchronous spike events in massively parallel spike train data.

In total, we use 6 different models of background activity, three with different levels of stationary rates and three with variable rates across times or neurons. We then vary, for each of these models, the number *z* of neurons involved in an injected STP and the number *c* of its repetitions from 3 to 10 in steps of 1, for a total of (*number of models* × *z* × *c*) = (6 × 8 × 8) = 384 parameter combinations. For each choice, we determine the performance of our approaches in terms of the average number of false positives (FPs) and false negatives (FNs), defined below, obtained over 100 stochastic realizations of the respective background model, yielding a total of 38,400 data sets to analyze.

### 3.3. False positives and false negatives

In pattern discovery, different definitions of false positives (FPs) and false negatives (FNs) are possible. The identification of the exact injected pattern is a clear example of correct identification (true positive, TP), while the identification of a pattern being completely disjoint from the injected pattern is a clear FP result. Similarly, the complete non-detection of an inserted pattern or subsets of it is an unambiguous FN outcome. Cases in between are less clear and need to be defined. For instance, the identification of a pattern whose spikes form a subset of the real pattern may be considered, depending on the portion of spikes of the found pattern relative to the true pattern, sufficient for a correct identification (TP). Here we adopt the most strict definition of a TP, i.e., classifying a found pattern as a TP if it consists of all and only the spikes forming each occurrence of the injected pattern. Otherwise, the pattern is detected as a FP and the absence of TPs yields an FN outcome.

We compute two types of FPs. One is based on purely independent data, i.e., we only realize the respective background model without pattern injection. This provides us the FP level which is purely resulting from the stochasticity of the processes. The second type of FPs we are exploring are STPs that were detected but were not the injected STPs. This is relevant if we want to make sure that injected patterns are not forming new patterns with the background activity. The results for the latter ones are shown in figures below, the former ones will be just mentioned.

For evaluation of FNs as a function of the signature (z,c) of the injected pattern, we vary the parameters *z, c* between 3, …, 10 to get all combinations. For each signature we perform *R* realizations of a given background rate model and insert a pattern of size *z* and with *c* occurrences. We evaluate in how many of the *R* realizations we detected the injected pattern. The resulting FN rate, i.e., the fraction of realizations in which we did not detect the pattern divided by *R*, is entered in a matrix at the signature *z* (x-axis) and *c* (y-axis). By varying the signatures and performing this procedure again we fill the FN matrix.

For evaluation of the FP matrix, we use the same data as for the FN evaluation. For each signature, we count the number of realizations in which one or more patterns are detected as significant that are not identical to the pattern injected. The ratio of the realizations for which this occurred divided by the number of realizations *R* is entered at the signature of the injected pattern.

In the next sections, we test the performance of our approaches in terms of FPs and FNs on our artificial, simulated test data.

### 3.4. Performance of approximate stability

**Figure 4:**
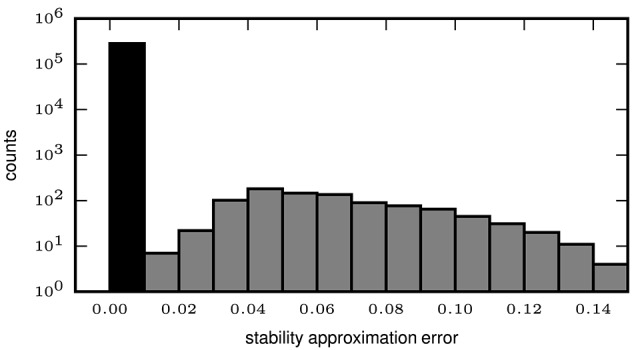
**Error of approximate calculation of intensional stability**. The histogram shows the number of patterns (in logarithmic scale) as a function of the absolute difference between the approximated and the exact intensional stability of one simulated data set. The leftmost black bar represents the number of patterns for which no difference occurs for the exact and the approximated stability which is the majority of the patterns (99.67%). The largest absolute errors (rightmost bar) are in the range of 0.13–0.14 and occur for 4 out of 283,451 total patterns. The average error (including also the error equal to 0, black bar) is 2.189 × 10^−4^.

In order to quantify the error introduced by the approximated stability (2.2.2), we compute the exact and the approximate intensional stability for all patterns extracted by the mining technique (FP-growth) from synthetic data. We set the number *Z* of subsets used for the Monte-Carlo approximation of the stability to 500, while for the computation of the exact stability all possible subsets are used. The data, already used in Yegenoglu et al. (2016), comprised parallel spike trains from 50 neurons firing independently of each other at a constant of rate *r* = 15 Hz each, for a total duration *T* = 1 s. In addition, we also generate data sets containing in addition an injected STP. The STP consists of *z* = 8 spikes from 8 different neurons, falling within a window of duration *w* = 50 ms. The STP is injected *c* = 9 times in the data, at random positions in the simulation period [0, *T*]. We define the approximation error as the absolute difference between the exact and the approximated stability values, both computed for each pattern extracted by FCA. The distribution of the errors greater than 0 is illustrated in Figure 4 (gray bars), with an average error of 1.888 ∗ 10^−3^ and a maximum error of 0.14 (i.e., 14% of the max. stability value). However, no errors at all (black bar in Figure 4) occur for the majority (282,510 out of 283,451, i.e., 99.67%) of the patterns. The results indicate that approximating the intensional stability is a suitable alternative to the computationally unaffordable calculation of exact stability and, thus, allows one to apply approximate stability of data sets of more than 50 neurons.

**Figure 5:**
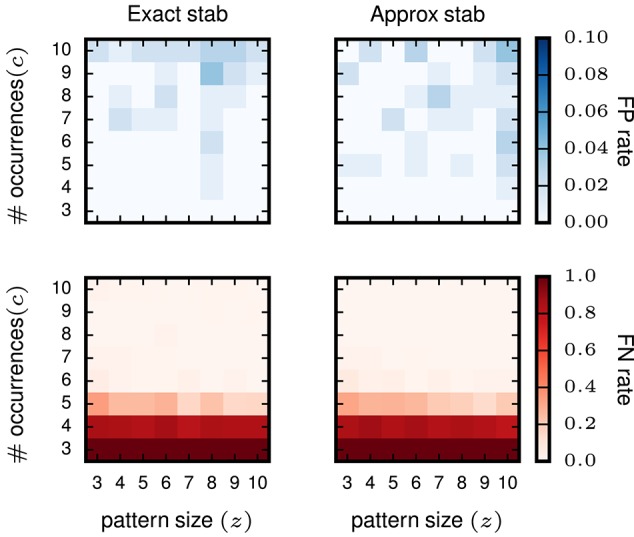
**Comparison of pattern selection based on exact and approximate stability**. The left column shows the FP matrix (top) and the FN matrix (bottom) for FCA analysis followed by exact stability filtering. The right column shows FP and FN after application of the approximate stability filter instead. For both approaches the threshold for the stability was > 0.6 for filtering the patterns. Each element of the matrices contains the FP or FN rate for a particular signature (*z, c*) of pattern size *z* (horizontal axis) and occurrence count *c* (vertical axis). The data analyzed are Poisson data (*N* = 50 neurons with stationary rate *r* = 15 Hz) with STPs injected with the respective signatures. *R* = 100 simulations are performed for FP/FN extraction.

Furthermore, we test whether or not the (small) error introduced by approximated stability does affect the performance of STP detection. To that end, we compare the results gained using exact stability and approximate stability to select significant concepts applied to the very same data. The data are also identical to the data analyzed in the previous study (Yegenoglu et al., 2016) composed of 50 neurons, simulated for 1 s with injected patterns with parameters *z, c* varying between 3 and 10, with 100 realizations for each parameter combination. Significantly stable concepts are detected if their stability crosses the threshold of θint = 0.6, i.e., the same as used in Yegenoglu et al. (2016). Figure 5 shows the results in terms of number of realizations returning FPs (top) and FNs (bottom) out of 100 simulations for each signature (*z, c*). The left column shows the results using exact stability, the right column using the approximated stability. The performances of both methods, both in terms of FPs and FNs, are qualitatively identical (maximum of the absolute value difference of the two matrices smaller than 0.1 for the FP-rate as well smaller than 0.5 for the FN-rate and randomly distributed across the matrix entries), showing that the error introduced by the Monte-Carlo approximation of the stability is negligible.

### 3.5. Validation on artificial data

We assess and compare now the performance in terms of FPs and FNs of the two variants of our analysis method, i.e., one that filters patterns on the basis of their stability and the other based on significance evaluation. We use the test data described in 3.2, i.e., data with a certain type of background activity (three different types with stationary rates, and three types with time-varying or inhomogeneous firing rates), and combined with injected STPs of a certain signature (*z, c*). For each data model, we generate *R* = 100 realizations (data sets), analyze each of them for the occurrence of STPs surviving the filtering process.

#### 3.5.1. Stability based filter results for stationary data

We first examine the performance of pattern filtering based on intensional (or extensional) stability. After choosing one of the two measures, this approach classifies patterns found by FP-growth as stable (and, thus, retains them as reliably reoccurring patterns) if their stability exceeds a pre-determined threshold θ. As explained in 2.2.4, we derive θ as a chosen quantile of the null distribution of stability values, obtained from independent data. We set the overall significance level to α = 0.01 and set θ to the percentile corresponding to the Bonferroni corrected level $\alpha_{\text{corr}}=\frac{\alpha }{\text{total number of concepts tested}}$.

To obtain stable patterns by application of the stability evaluation we make use of surrogate data, i.e., independent data generated by dithering (see 2.2.4) from the original data, to derive the null distribution and, thus, the stability threshold θ. For our extensive validation of data containing injected STPs we would have to derive the stability threshold for each of the total 100 × 6 × 8 × 8 = 38, 400 (see Section 3.2) data sets. To avoid such massive computations, we make use of the assumption that the few additional spikes injected by insertion of STPs do not change the null distribution of the stability values under the hypothesis of independence. Therefore, to evaluate FPs and FNs across all these scenarios, we derive a single stability threshold θ for all models with the same background rate as follows: we generate 100 data sets with independent background activity according to the rate model, and derive θ as the 95% quantile of the empirical distribution of pattern stability values in this case where no patterns were inserted. This threshold is then used for the assessment of FPs and FNs in all 64 models with the same background rate but containing STPs of different size and occurrence count. This approach, was already used in Torre et al. (2013) and was shown to be appropriate (see Yegenoglu et al., 2016), since the distribution of θ is not affected by the insertion of a few STP spikes. In addition, this approach makes our validation considerably (64 times) faster.

##### 3.5.1.1. Performance of intensional and extensional stability

We first analyze stationary data with constant firing rates, *r* ∈ {15, 20, 25 Hz} containing injected STPs. We filter the patterns found by FP-growth on the basis of either their intensional or their extensional stability, and calculate the corresponding significance thresholds θint and θext as explained above. In the following, only the results for *r* = 25 Hz are shown, as those for *r* = 15 Hz and *r* = 20 Hz are comparable and lead to identical conclusions. The FPs on purely independent data sets (only background activity) as defined in 3.3 has a FP rate of 0.01, i.e., in only 1% of the total realizations of the independent data FPs are detected (not shown).

**Figure 6:**
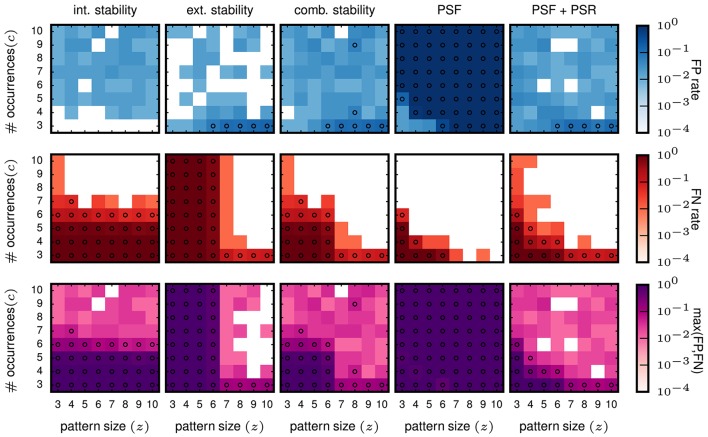
**Performance in terms of FPs and FNs for stationary data**. The results shown here are all for the data model in which all neurons have a stationary firing rate of 25 Hz. All data sets consist of 100 neurons simulated for 1 s. The parameters used for FP-growth are a bin size of 1 ms and a window length of 50 ms. We show the results for all types of injected patterns entered at their respective signature [64 signatures, all possible combinations for the size *z* (x-axis) and the number of occurrences *c* (y-axis)]. Size and number of occurrences are varied between 3 and 10. For each pattern signature we perform and analyze 100 realizations. Each matrix element (signature) shows the fraction of realizations for which the filtered results contain one or more FPs (top row) / FNs (middle row). The bottom row shows the maximum rate of either the FPs or the FNs. *First column:* Results of the intensional stability filter, using a significance level of α = 0.01 and Bonferroni corrected, yielding a stability threshold of θint ≈ 0.55*. Second column:* Results of the extensional stability filter and stability threshold of θ_*ext*_ ≈ 0.8 resulting from the same significance level as for the intensional stability. *Third column:* Results of the combined stability filter, where the Bonferroni correction was adjusted by a factor 2 to account for each concept being tested twice (once for the extensional and once for the intensional stability). *Fourth column*: Results of PSF. Significance level α = 0.01, corrected with the FDR criterion. *Fifth column:* Results of PSF+PSR. Significance level α = 0.01, corrected with the FDR criterion. The PSR parameters are set to *h* = 0, *k* = 2.

Then we evaluate the performances for data with injected patterns. In the columns on the left of Figure 6, we show the FP and FN matrix for the analysis using intensional and extensional stability, respectively. Each entry in the matrices corresponds to one signature (*z, c*) of the injected pattern, and the color-coded value represents the FP (top panels) or FN (middle panels) rate. The FP rates for both, the intensional (left column) and the extensional stability (second column from left), are low, but somewhat higher (some times at 0.05, marked by circles in Figure 6) than for FPs in purely independent data.

The FN rates (Figure 6, middle row) are large (often close to maximum) for about half of the matrix elements for both stability measures but differ in respect to the signatures at which they occur. For intensional stability, the FNs are large for the whole range of *z*, but only for small *c* (about 3–6), and decrease abruptly for larger c. In contrast, when filtering with extensional stability, the FNs are high for all *c*, but only for small *z* (about 3-6) and decrease for larger *z*. These results are not unexpected since, as explained in 2.2, intensional stability is almost exclusively affected by the number of occurrences of a pattern, while extensional stability is emphasizing the number of spikes forming a pattern. Nevertheless, these are undesired results, since in independent data chance patterns decay with their number of occurrence and the pattern size (see e.g., Torre et al., 2013) and, thus, we expect that the border of selected patterns should also decay as a function of the combination of both parameters.

##### 3.5.1.2. Combined stability

Aiming at a method whose FNs decay with the size *z* and the occurrence count *c* of the patterns, we combine the filtering criteria based on intensional and extensional stability. This approach keeps all concepts whose intensional or extensional stability value is larger than the respective thresholds. This procedure applied to independent data leads to a maximum FP rate equal to 0.02 (not shown), and can be explained by the application of two tests on the data. The results for the data sets containing the STPs are shown in the third column from left in Figure 6. The FP rate is again smaller than 0.05 for most of the entries, and higher than 0.05 for entries in the right bottom corner of the matrix, which result from data sets that contain patterns of large size occurring only 3 times and easily combine with background spikes and, therefore, become significant. The FNs instead decrease gradually, both as a function of pattern size *z* and of the pattern count *c*. Thus, the combined filter retains large patterns due to their high extensional stability, and patterns with a large occurrence count due their high intensional stability.

#### 3.5.2. Significance based filter results for stationary data

For further comparison we analyze the same data as above using FP-growth followed by PSF and PSR. We set the significance level for the PSF to α = 0.01, corrected by the number of different pattern signatures in the data using FDR correction. Note that when setting the threshold for extensional and intensional stability we use the more conservative Bonferroni correction instead, because FDR did not provide an adequate compensation (i.e., it leads to a large number of FPs, not shown here).

The FP rate of independent data is as for the stability filtering smaller or equal to 0.01 (not shown). In the independent data, PSF alone suffices to achieve this performance. PSR is not necessary, because the probability that completely chance patterns exceed the PSF significance threshold and overlap is close to 0. In contrast, PSR is a critical step for data containing non-chance patterns, where it is designed to remove the false positives found by PSF due to the overlap of the true patterns with the background activity. Without correcting for overlapping patterns the results show very high FP rates for any combination of size and number of occurrences of the patterns (Figure 6, fourth column, top). Using PSR the FP rates for data with injected patterns (Figure 6, right column, top) are similarly low as for the other approaches, i.e., at the level of 0.05. Some FPs have a FP rate larger than 0.05, and occur for data containing injected patterns with large size and small number of occurrences (*c* = 3). As for the other methods, these FPs are due to combinations of injected patterns and background activity. The FNs decay as a function of *z* and *c*, but much faster than the combined stability approach (Figure 6, right column, middle). The PSR moderately increases the number of FNs for patterns with few neurons occurring often or composed by many spikes and occurring few times. This is due to the fact that these are the two conditions in which it is more likely to have one or more spikes of the noise background that overlap by chance with the injected pattern forming a larger, more significant pattern. Such chance overlap may cause the rejection of the injected pattern in the PSR.

Counting the number of STPs obtained after each step of the of the method clarifies the impact of that step on the overall performance. While these numbers change in magnitude depending on the parameters (*z, c*) of the injected pattern, their proportion between different steps of the method was very similar across different values of (*z, c*). We can, therefore, illustrate the results for one specific configuration (*z* = 10, *c* = 10). Each step is maximally effective if the number of STPs it keeps as non-chance patterns is 1 and if this pattern is the single injected pattern. For *z* = *c* = 10, we obtained on average: (a) 1089.92 STPs after FIM (which retains all frequent closed patterns), (b) 2 STPs after either the combined or the intensional-only filtering, and 1 STP after extensional-only filtering, (c) 24.41 STPs after PSF, and (d) 1.02 STPs after PSF+PSR (almost exclusively the injected pattern).

In conclusion, based on the validation of stationary data, the approach based on PSF and PSR, i.e., the SPADE method extended to spatio-temporal patterns, performs best and has the highest detection power as compared to the methods based on stability analysis. Even the combined stability analysis that uses the two stability measures independently has a smaller range of signatures with low FNs and improves only if at least one of the patterns' parameters *z* or *c* is large enough. In contrast, SPADE is also sensitive to the total number of spikes (*z* × *c*) in the patterns. Thus, both approaches (stability or significance based) show the desired feature of (a) small number of FPs, (b) decreasing number of FNs for increasing (*z, c*). However, SPADE produces a smaller total number of FNs for comparable FP rate and, thus, has the best performance. We finally note that the patterns found by extensional stability filtering were almost always found by PSF+PSR too (which is slightly less conservative, and which was less prone to false negatives). Thus, combinations of these two selection criteria do not seem a valid option here. For instance, retaining all patterns found by any of the two criteria would be often identical to accepting the results from PSR+PSR. Retaining only patterns found by both would most of the times be equivalent to accepting the results from extensional stability. Both options would additionally sum the computational costs of the two methodologies.

#### 3.5.3. Performance of SPADE in the presence of multiple STPs

The experiments illustrated so far were performed on data containing a single true STP, which the method was able to find with high reliability. Real data, however, are likely to contain STPs from more than one group of neurons. Experimental studies (e.g., Riehle et al., 1997; Torre et al., 2016b) revealed, for example, an abundance of synchronous spike patterns arising during task execution. Torre et al. (2016b) used the original version of SPADE, demonstrating its ability to retrieve multiple synchronous patterns, when present. To demonstrate that our extended method can achieve the same for STPs, we investigated an additional scenario with data containing two different types of injected STPs. Both STPs had size *z* = 5, were injected *c* = 10 times, and had an inter-spike delay of 5 ms. We generated 100 realizations of this model. At each iteration, the neurons involved in each STP were selected randomly, but such that they would not form two identical sets. We obtained no FPs and no FNs. For the realizations where the two patterns overlapped, PSR successfully retrieved them, while correctly rejecting their intersection as a FP. This demonstrates that SPADE can indeed cope well with complex scenarios entailing multiple, even overlapping STPs.

### 3.6. Validation of SPADE on inhomogeneous data

**Figure 7:**
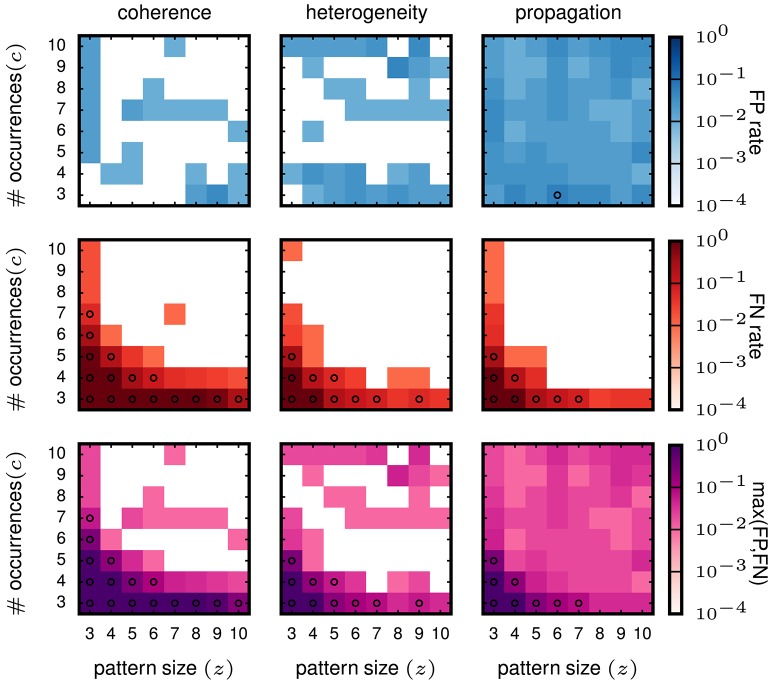
**Performance of SPADE on non-stationary and inhomogeneous data**. SPADE is applied to stochastic simulations of different rate data models, left column: rate coherence, middle column: rate heterogeneity, right column: rate propagation. For the FP rate (top row) and FN rate evaluation (middle row), patterns of a given size *z* and number of occurrences *c* are inserted into the same background rates (given by the data model). In the bottom row, the maximum of FP or FN of each (z,c) signature is shown. The same settings are used for FP-growth and SPADE as in Figure 6.

From the validation on stationary data we conclude that SPADE performs better than filtering methods based on stability measures. Therefore, we now concentrate on the SPADE method only. We aim at evaluating the performance of SPADE on data that mimic more closely features of experimental data, such as non-stationarity of firing rates in time and inhomogeneous firing rates across neurons. In particular, we study three cases (firing rate co-modulation, rate hetereogeneity across neurons, and rate change propagation, as introduced in 3.2) that are known to be potentially strong generators of false positives for correlation analysis methods for (see e.g., Grün et al., 2003; Grün, 2009; Louis et al., 2010; Torre et al., 2016a). The results are shown in Figure 7.

The FP rate of the analysis of data with injected pattern is generally low (less than 0.05), for all three types of data. For firing rate co-modulation and rate inhomogeneity the FP rate is often virtually zero (white squares) but is somewhat more homogeneously at 0.05 for rate change propagation. This is somewhat surprising that such successive increases of firing rate, occurring repeatedly, do not elicit a higher level of FPs. The FNs decay with *z* and *c* in all three scenarios and fastest for the rate propagation model. Interestingly, these results are not worse than for the stationary data, meaning that SPADE can deal well with data that contain features that are typically generating FPs. In conclusion, SPADE can tolerate coherent rate non-stationarities and inhomogeneous rates and, thus, is qualified to be applied to experimental data.

### 3.7. Summary of the validation results

Our validations highlight the following aspects:

- fast-FCA and FP-growth lead to identical mining results, however, the better computational performance of FP-growth allows one to mine concepts of real-sized data (100 or more neurons recorded over several seconds).
- Stability (approximate) filtering and significance filtering (the combination of PSF and PSR, i.e., SPADE) are efficient statistical techniques to reject chance patterns in independent (STP-free) data, as they all exhibit a small FP rate (<1% for PSF+PSR, intensional stability and extensional stability; <2% for the combined stability filter).
- FPs on data with injected patterns shows that all methods perform about the same with FPs on the 5% level.
- Significance filtering (SPADE) is the technique that best detects injected STPs in the data, exhibiting a FN rate below 5% for patterns with lower size or occurrence counts than stability filtering.
- These considerations for SPADE also hold for data with highly variable firing rates over time and across neurons, which suggests that the combination of FP-growth, PSF and PSR is the best technique to detect STP in real recordings.
- At the same time the stability based analyses, which have an equally low FP rate, although less sensitive to the presence of STPs, is applicable to particularly long recordings for which PSF is computationally not feasible.

## 4. Discussion

The ever growing number of neurons that modern electrophysiological techniques allow to record in parallel provides access to the coordinated spiking activity of neuronal populations of unprecedented size. The investigation of millisecond-precise spatio-temporal spike patterns (STPs) in large scale recordings becomes, therefore, possible. However, suitable analysis techniques have been lacking so far due to the exponential growth of the number of STPs in such large data, which yields severe computational and multiple testing issues.

Here we addressed this problem by introducing a method, named SPADE (spatio-temporal Spike PAttern Detection and Evaluation), that extracts STPs from massively parallel spike train data and assesses their statistical significance under the hypothesis of spike independence. SPADE builds on and brings together two techniques that we had previously introduced for the identification of STPs in massively parallel spike trains (Yegenoglu et al., 2016) and for the statistical evaluation of patterns of synchronous spikes (Torre et al., 2013). The latter avoided the computational and multiple testing issues that usually prevent applying such analyses to large data sets. The underlying pattern mining algorithm FP-growth, however, was implemented such that the technique was applicable for the discovery of synchronous spike patterns only. A restructuring of the input data format (“attribute scaling” in the language of FCA, see Ganter and Wille, 1999) allowed us now to use FP-growth (or similar frequent pattern mining techniques, see e.g., Borgelt, 2012) to extract more general STPs. Thus, FP-growth served here the same purpose that fast-FCA served in Yegenoglu et al. (2016), that is, counting the occurrences of non trivial repeating patterns (there named “intents”). As known from the literature (Zaki and Ogihara, 1998) FIM and FCA yield results that can be mapped one to one onto each other: they extract closed frequent itemsets / formal concept intents including their occurrence count. Our implementation of FP-growth, however, proved to be much faster than the state-of-the-art implementations of the FCA algorithms available to our knowledge. Soon, a C implementation of the FCA's In-Close algorithm (Andrews, 2009) will be made available by S. Andrews, N. Kodoga and colleagues, which may provide a mathematically equivalent but computationally even faster algorithm to mine re-occurring STPs.

SPADE assesses the significance of the patterns identified by FP-growth or equivalent algorithms via the same analysis steps as in Torre et al. (2013). First, pattern spectrum filtering (PSF) is used to determine the *p*-value of signatures (*z, c*), i.e., pairs of pattern size *z* and occurrence count *c*, and retains patterns with significant signatures only. The number of different signatures in data is typically orders of magnitude smaller than the number of different patterns. Thus, testing for the signatures reduces the multiple testing problem to a size that can be handled with standard statistical corrections, such as false discovery rate. Then, pattern set reduction (PSR) is applied to test all patterns identified by PSF, conditioned on the presence of any other pattern in the remaining list. This allows one to distinguish, among overlapping patterns, the genuine ones from those that can be explained as a chance overlap of real patterns with background spikes. Validation on test data generated by different stochastic models of STPs injected into background activity demonstrated the ability of the method to discriminate real and chance STPs, ensuring low false negative (FN) and low false positive (FP) levels despite the large number of STPs to test (up to millions). For example, in simulated data consisting of 100 neurons spiking independently at an average rate of 15 Hz each for a period of 1 s, an injected STP was successfully isolated from the background activity as soon as it involved at least 5 neurons and it repeated 3 times, or it involved as low as 3 neurons and repeated 5 or more times. The method showed high power (FN rate lower than 5%) and reliability (FP rate lower than 5%) in different scenarios replicating various features of the firing rates often observed in experimental data, which typically represent strong generators of FPs (Louis et al., 2010). These include abrupt and coherent rate changes over time, largely different firing rates across neurons, sudden rate changes propagating from one group of neurons to the other. Our method performs well in all of these scenarios.

Besides qualifying STPs as excess patterns on the basis of their statistical significance, we additionally explored various ways to extract them from background activity on the basis of their extensional or intensional stability. In FCA terminology, the extent of a concept in our context (frequent closed pattern) is the set of windows the patterns falls into. The intent of a concept is its composition, i.e., the neuron index and time index (within the window) of each composing spike. Intensional stability quantifies the tendency of a pattern occurring in a set of windows to be the largest pattern common to those windows (or any subset thereof). Low intensional stability indicates that the intersection of any number of those windows tends to contain supersets of that pattern and, therefore, that the pattern occurrences may have been the result of intersections of fewer occurrences of different larger patterns. Similarly, extensional stability quantifies the tendency of a set of windows to contain a subpattern of the pattern which comprises its intent, such that the subpattern is not found in any window that does not contain the pattern. Intensional and extensional stability are used as indicators of how reliably the pattern can be considered as a genuine event, rather than the sum of occurrences of larger patterns or the superposition of smaller patterns occurring in the same time segments, respectively. In Yegenoglu et al. (2016) we explored the use of intensional stability to isolate reliably re-occurring STPs from high background activity. The exact computation of the stability of each pattern, however, is computationally very demanding, and was possible only on data comprising a maximum of 50 neurons simulated for a few seconds. Here we adopted the Monte-Carlo based approximation of stability introduced by Babin and Kuznetsov (2012), which allowed us to speed up the computation by several orders of magnitude while introducing negligible errors and, thus, enabled the application of intensional as well as extensional stability based pattern filtering to larger data. In particular, we computed a statistical threshold for both intensional and extensional pattern stability, using independent surrogate data, and we filtered out patterns whose (intensional, extensional, or both) stability values were lower than the respective thresholds. Compared to the previous approach based on pattern significance, all of these stability-based criteria were computationally less demanding but yielded increased FNs, especially when the injected STP to be retrieved had low pattern size. Nevertheless, other combinations of these approaches may be envisioned in the future to improve the performance of the method even further if even more spike trains become available in parallel.

Existing methods for the identification of repeating STPs are either not applicable to data sets of large size, or limit the search to patterns with specific features (of fixed, usually small size, or exhibiting specific inter-spikes intervals such as synchronous patterns). SPADE does not suffer from these limitations thanks to a combination of attribute scaling, fast frequent item set mining, and a hierarchy of tests of pattern significance, which avoids severe multiple testing. Its extensive validation ensures its reliability in applications to real data, as well as to simulated data resulting from network models. SPADE can be applied not only to spike data, but also to any data consisting of parallel point processes, such as discretized calcium imaging data (e.g., Roth et al., 2012), discretized voltage-sensitive dye imaging data (Ayzenshtat et al., 2010) or discretized MEG recordings (e.g., see Tal and Abeles, 2016). In the work of Ayzenshtat et al. (2010) and Tal and Abeles (2016) the authors defined and extracted special events from their analog recordings (voltage-sensitive dye imaging and MEG, respectively) and reduced them to point events. In the published work they analyzed subsets of the resulting parallel point processes for pair-wise of triple-wise correlations or spatio-temporal patterns, respectively, and identified those in relation to the behavior. With SPADE the complete data sets from these recordings (i.e., massively parallel point processes) could be analyzed. The analysis time scale is thereby not restricted to milliseconds (as employed here) but can be freely adjusted depending on the research question. The analysis time scale is not restricted to milliseconds (as employed here) but can be freely adjusted depending on the research question.

## Author contributions

SG, DE, and ET designed the work. PQ, AY, ET, DE, SG designed the analysis and statistical tools. PQ, AY, ET, DE wrote the analysis software and performed the experiments; All authors contributed to the writing of the manuscript.

### Conflict of interest statement

The authors declare that the research was conducted in the absence of any commercial or financial relationships that could be construed as a potential conflict of interest.
